# PPARγ Promotes Urothelial Remodeling During Urinary Tract Obstruction

**DOI:** 10.21203/rs.3.rs-5234950/v1

**Published:** 2024-12-02

**Authors:** Ashley Jackson, Mohammad El-Harakeh, Felipe Rodriguez-Tirado, Alexa Miehls, Andrew Hardman, Oumoulkhairy Camara, Kelly Grounds, Glenis Tocaj, Macie Kercsmar, Birong Li, Xin Wang, Brian Becknell

**Affiliations:** Nationwide Children’s Hospital; Nationwide Children’s Hospital; Nationwide Children’s Hospital; Nationwide Children’s Hospital; Nationwide Children’s Hospital; Nationwide Children’s Hospital; Nationwide Children’s Hospital; Nationwide Children’s Hospital; Nationwide Children’s Hospital; Nationwide Children’s Hospital; The Abigail Wexner Research Institute at Nationwide Children’s hospital; Nationwide Children’s Hospital

## Abstract

Urinary tract obstruction (UTO) is a common cause of kidney injury that can result in chronic kidney disease and end-stage renal disease. Heterogeneity in the extent of obstructive renal damage in humans with UTO implies the existence of unknown mechanisms that protect against or accelerate kidney injury. Prior studies show that congenital and acquired UTO initiate a conserved, protective program of renal urothelium remodeling that culminates in expansion of uroplakin (UPK)+ cells to promote renal structural integrity. However, the cellular and molecular mechanisms that regulate UPK expression in the renal urothelium are unknown. Peroxisome proliferator-activated receptor γ (PPARγ) drives urothelial differentiation and UPK expression in other tissues but has not been investigated in the renal urothelium. Here, we demonstrate that activation of PPARγ in UPK+ cells is critical for UTO-induced renal urothelium remodeling. Conditional deletion of PPARγ perturbs UPK expression and accelerates parenchymal thinning during UTO, while conditional activation of PPARγ increases UPK expression and results in parenchymal preservation. This study underscores the significance of renal urothelium during UTO and shows that UTO-induced renal urothelial remodeling is achieved through activation of PPARγ. These findings form the foundation for future studies that will determine the therapeutic utility of PPARγ agonists during congenital and acquired UTO.

## Introduction

Urinary tract obstruction (**UTO**) is a common cause of kidney injury that can lead to acute kidney injury and chronic kidney disease^1–3^. Acquired forms of UTO predominate in adults, while congenital etiologies predominate in children. Beyond interventions to relieve UTO, there are no available measures to prevent or reverse obstructive kidney injury. Research designed to improve UTO outcomes has been prioritized in the long-term strategic plans of major urological organizations^4,5^.

Prior studies show that congenital and acquired UTO initiate a conserved, protective program of renal urothelium remodeling that culminates in expansion of uroplakin (**UPK**)+ cells^6–8^. Renal UPK+ cells express UPK1A, UPK1B, UPK2, and UPK3A, which assemble immature urothelial plaques at the apical surface during homeostasis^9^. Following obstruction, UPK+ cells form mature, bladder-like urothelial plaques known to contribute to the formation of a compliant and impermeable urothelial barrier^10–12^. Depletion of UPK+ cells or ablation of the urothelial plaques using *Upk1b*-null mice accelerates parenchymal loss, renal dysfunction, and mortality during UTO^7^. Therefore, UPK+ cells promote renal structural integrity during congenital and acquired UTO^7^. This suggests that efforts to drive UPK expression may attenuate obstructive kidney disease, but the cellular and molecular mechanisms that regulate UPK expression in the renal urothelium are unclear.

Notably, renal urothelium is morphologically distinct from renal pelvis, ureter and bladder urothelium^13,14^, and renal urothelium cell types and distributions are unique^15^. Moreover, the developmental origin of UPK+ cells in renal urothelium is distinct from lineage relationships observed in bladder and ureteral urothelium^15–18^. In the kidney, keratin-5 (**KRT5**)+ cells exhibit an age-restricted potential to form UPK+ cells^15^. While, UTO-induced urothelial injury widens the window of multipotency, adult KRT5+ cells appear unable to escape lineage restriction and do not form the majority of UPK+ cells following UTO^15^. Thus, the adult renal urothelium repair progenitor remains unknown. In the bladder, adult intermediate UPK+ cells function as tissue repair progenitors^17,19,20^. The existence of an intermediate-like UPK+ cell in the kidney is uncertain, and a progenitor role for UPK+ cells in the kidney has not been investigated.

Peroxisome proliferator–activated receptor γ (**PPARγ**), is a nuclear receptor associated with transcription of genes linked to differentiation in numerous cell types, including urothelium^21–27^. Upon ligand binding, PPARγ heterodimerizes with retinoid X receptor a (**RXRa**), associates with cofactors and binds to peroxisome proliferator response elements (**PPRE**) on target genes, such as fatty acid-binding protein 4 (**FABP4**)^22,28^. Pharmacologic activation of PPARγ directs urothelial differentiation in 2D and 3D cultures^23,26^. PPARγ indirectly promotes UPK (UPK1A, UPK2, and UPK3A) expression through regulation of intermediary transcription factors, forkhead box A1 (**FOXA1**), interferon regulatory factor-1 (**IRF-1**), and grainyhead-like 3 (**GRHL3**)^22,25,29^.

Recent *in vivo* studies demonstrate that conditional deletion of PPARγ in urothelial cells abrogates the formation of terminally differentiated bladder urothelial cells, while conditional activation of PPARγ promotes differentiation of urothelial basal cells^21,22^. Although UTO engages a program of renal urothelium differentiation, a role for PPARγ in the renal urothelium or during UTO-induced renal urothelium remodeling has not been investigated.

In this study, we used lineage analysis to show that adult UPK+ cells serve as tissue repair progenitors during UTO and found that UTO induces PPARγ expression and activation in UPK+ cells. Conditional deletion of PPARγ during UTO perturbs UPK expression and accelerates parenchymal thinning, while conditional activation of PPARγ enhances UPK expression and results in parenchymal preservation during UTO. These findings form the basis for the development of therapies aimed at bolstering the renal urothelium to mitigate obstructive kidney disease.

## Materials and Methods

### Animals

Public Health Service Animal Welfare Assurance number A3544–01 and Institutional Animal Care and Use Committee number AR16–00058 were used. Tg(Upk2-icre/ERT2)1Ccc/J (*Upk2*^CreERT2^, Jax #024768)^30^, B6.Cg-*Gt(ROSA)26Sortm14(CAG-tdTomato)Hze*/J (*R26*^tdT^, Jax #007914)^31^, B6.129-*Pparg*^*tm2Rev*^*/*J (*Pparg*^fl/fl^, Jax #004584)^31^, and *ROSA26-CAG-STOP-VP16PPARG-IRES-EGFP (VP16*-*Pparg*^fl/fl^)^32^ mice were used (**Supplementary Table 1**). *Genotyping*. Tail DNA was isolated for genotyping by standard PCR (**Supplementary Table 1**). The order in which surgery, ultrasound, and euthanasia were performed in control and mutant mouse groups was random to minimize potential confounders. The surgeon, ultrasonographer and downstream analytical team were blinded to mouse genotypes and group allocation to minimize bias.

### Histology and Immunofluorescence

Formalin-fixed tissues were paraffin processed, sectioned at 4 microns, and mounted on charged slides. Routine hematoxylin and eosin (**H&E**) and Picrosirius Red (**PSR**) staining were performed. Immunolocalization was performed using anti-PPARγ (Cell Signaling Technology, Danvers, MA), anti-FABP4 (R&D Systems, Minneapolis, MN), anti-FOXA1 (also called HNF4, Santa Cruz Biotechnology, Dallas, TX), anti-GRHL3 (Abcam, Boston, MA) anti-KRT5 (BioLegend, San Diego, CA; Abcam Inc., Waltham, MA), anti-RXRa (Cell signaling Technology, Danvers, MA), anti-TDT (tomato, Rockland Immunochemicals, Limerick, PA; MyBioSource, San Diego, CA), anti-UPK1A (Santa Cruz Biotechnology), anti-UPK1B (Sigma-Aldrich, St. Louis, MO) and anti-UPK3A (AbClonal, Woburn, MA; Fitzgerald, Acton, MA) primary antibodies (**Supplementary Table 2**). Cy3, Cy5 and Alexa Fluor 488 secondary antibodies (Jackson ImmunoResearch Laboratories, West Grove, PA) were used at 1:300. Coverslips were mounted using ProLong Antifade (Fisher Scientific, Hampton, NH). Images were captured using a Nikon Ti2-E microscope and ORCA-Fusion GenIII camera (fluorescent micrographs) or DS-Ri2 color camera (brightfield and polarized micrographs) (Nikon Instruments, Inc., Melville, NY). Micrographs underwent equivalent brightness and contrast adjustments to enhance print view. Results were quantified using ImageJ v1.53k, or QuPath v0.4.3 open source software^33^. *Fluorescent image analysis*: Briefly, the renal urothelium was manually annotated using QuPath, and cell detection or pixel thresholding tools were applied, and images were batch processed. *PSR image analysis*: Briefly, ImageJ was used to annotate regions of interest (ROIs) on PSR images collected using standard brightfield images (5–11, 20X images/kidney). The suburothelium compartment was annotated as a ROI spanning 100 mm beneath the renal urothelium. The renal cortex and medulla were captured as 20X images. ROIs were transferred to polarized images, and the color threshold tool was used to annotate collagen fibers (spanning red, green, yellow hues). All measurements were exported to excel then added to GraphPad Prism v10.1.1 to apply appropriate statistical analyses as indicated in figure legends (GraphPad Software, Boston, MA).

### Cre Recombination

Tamoxifen (TMX, Sigma Aldrich) was dosed *via* intraperitoneal (**ip**) administration at stages defined in each experiment to induce Cre-mediated recombination. For lineage experiments (using R26^tdT^ line), a single dose of TMX (75 mg/kg body weight [**b.w.**] in corn oil) was administered. For conditional deletion or conditional activation of PPARγ (using *Pparg*^fl/fl^ and VP16-*Pparg*^fl/fl^ lines), TMX (37.5 mg/kg b.w.) was administered six times over two weeks leading up to surgical obstruction, followed by two doses following obstruction. Unexpected TMX effects were ruled out by using Cre-negative mice, and inappropriate Cre;LoxP recombination was ruled out by using corn oil (carrier) treatment.

### Unilateral Ureteral Obstruction (UUO)

UUO was performed according to methods taught at the Mouse Kidney Injury Workshop (Vanderbilt University Medical Center, Nashville, TN) as previously described^7,15^. Briefly, following inhalation isoflurane induction, the right kidney was dorsally externalized, and the proximal ureter was ligated using a nonabsorbable silk suture. Ureteral ligation was omitted for sham surgery. The surgical site was closed using absorbable PDS II suture and buprenorphine (0.05 mg/kg ip) was administered for analgesia. UUO was performed by the same experienced member to minimize variability. Every effort was made to reduce pain, suffering and distress. Humane endpoints established at IACUC review were strictly followed and mice were monitored daily. UUO was well tolerated and none of the mice in this study met endpoint criteria.

### Renal Ultrasound

Renal ultrasound was performed and hydronephrosis was graded as previously described^6,7,9,34^. Briefly, isoflurane anesthetized mice were shaved and depilated. Using a 40-mHz transducer, the longitudinal axis of each kidney was visualized. The largest longitudinal plane was imaged and the percent parenchyma was calculated using the following equation: ([transverse renal width – renal pelvis diameter] + renal papilla width)/transverse renal width^34^.

### RNA extraction, cDNA synthesis, and qPCR

RNA was extracted from decapsulated, snap-frozen whole kidneys using the mirVana kit (Life Technologies, Carlsbad, CA) as previously described^6,7,9^. RNA was reverse transcribed to cDNA using the Thermo Scientific^™^ Verso cDNA Synthesis Kit (Fisher Scientific). *Power* SYBR^™^ green PCR master mix (Fischer Scientific) and gene-specific primers (**Supplementary Table 3**) were used to amplify 50 ng of cDNA on a 7500 real time PCR system (Applied Biosystems, Waltham, MA). Results were expressed using the 2^−ΔΔCT^ method normalized to the housekeeping gene *Gapdh* (**Supplementary Table 3**). Results were graphed and analyzed using GraphPad Prism.

### Single cell RNA-seq (scRNA-seq) data collection and analyses

Single-cell combinatorial indexing RNA sequencing (sci-RNA-seq3) datasets from mouse kidneys were obtained from the Gene Expression Omnibus (GEO) database under accession number GSE190887^35^. The datasets encompassed samples collected at six time points: 0, 2, 4, 6, 10, and 14 days post-UUO representing both UUO-affected and healthy (called baseline) kidneys. A total of 162,611 high-quality cells were collected in the analysis, covering 19 distinct kidney cell types. To assess the effects of UUO on kidney cellular populations, we systematically compared the expression of key urothelial markers across all cell types between UUO and control conditions.

For tracing the UPK-lineage, we extracted cells belonging to the urothelial cell population expressing at least two read counts of any of the following UPK markers: *Upk1a, Upk1b, Upk2*, and *Upk3a*. This selection resulted in 1,274 UPK+ cells, including 318 from baseline kidneys and 956 from UUO-affected kidneys. The extracted UPK+ cells were processed following the standard Seurat workflow. Cells were normalized using SCTransform, a method that normalizes and stabilizes variance across cells while accounting for sequencing depth. We identified the top 2,000 most variable genes across the dataset, which were used for downstream analysis. To mitigate technical noise, including mitochondrial gene expression variability, we regressed out the percentage of mitochondrial gene content during the scaling process. Principal component analysis (**PCA**) was performed on the scaled data to reduce dimensionality and identify key sources of variation within the dataset. The optimal number of principal components was determined using an elbow plot. To account for batch effects arising from different time points and sample conditions, we applied Harmony, a batch correction algorithm, to ensure robust alignment of cells across conditions without compromising biological variance. Dimensionality reduction was performed using Uniform Manifold Approximation and Projection (**UMAP**). Clustering was conducted using the Louvain algorithm through Seurat’s FindNeighbors and FindClusters functions, with the clustering resolution empirically determined to balance granularity with biological interpretability. UMAP plots were generated to visualize the cellular landscape and identify distinct subpopulations within UPK+ cells.

To identify differentially expressed genes (**DEGs**) in UPK+ cells between baseline (POD0) and UUO conditions, we utilized the “MAST” implemented in Seurat’s FindMarkers function. Genes were considered differentially expressed if they met the following criteria: an adjusted p-value £ 0.05 (Bonferroni correction) and a fold change ≥ 1.5. DEGs were functionally annotated through Gene Ontology (**GO**) enrichment analysis using ClusterProfiler. Additionally, we used ChIP-X Enrichment Analysis (**ChEA3**) to predict transcription factors regulating the observed DEGs in the UPK+ cells during UUO.

### Statistical Analyses

Statistical analyses were performed using Prism Software. Normality was tested using the Shapiro-Wilk test. When appropriate, we applied a One Way Anova, an unpaired two-tailed *t* test, or unpaired Mann-Whitney test. Differences between groups with a *P* value *P* < 0.05 were considered statistically significant. Data are presented as means ± SD. Figure legends indicate sample size, sex, statistical tests and relevant multiple comparison correction used.

## Results

### Adult UPK+ cells contribute to renal urothelium remodeling during UUO.

**Figure 1:**
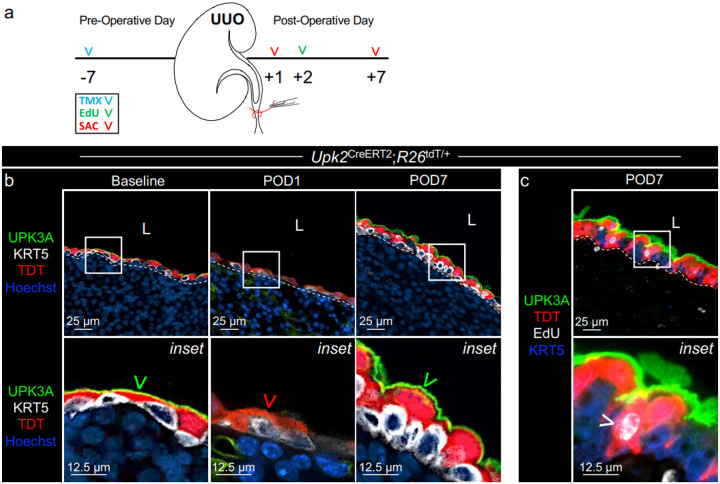
Adult UPK+ cells contribute to renal urothelium remodeling during UUO. **a** Schematic depicts experimental plan. UUO: Unilateral Ureteral Obstruction; TMX: Tamoxifen; SAC: Sacrifice. **b** Representative micrographs and insets show anti-UPK3A, -KRT5, -TDT, and Hoechst in the renal urothelium of Upk2CreERT2;R26tdT/+ mice at baseline, post-operative day (POD)1, and POD7. **c** Representative micrograph and inset shows anti-UPK3A, -KRT5 -TDT and EdU labeling in the renal urothelium of Upk2CreERT2;R26tdT/+ mice at POD7. Notably, EdU was dosed at POD2. White dashed line: renal urothelium basement membrane; L: lumen; Green arrowhead: TDT+ UPK+ cell; Red arrowhead: TDT+ cell with undetectable UPK3A expression. White arrowhead: EdU+ TDT+ UPK+ cell. Upk2CreERT2;R26tdT/+ (n=4 mice).

UUO triggers a sequence of renal urothelium remodeling that includes 1) loss of UPK expression at post-operative day (POD)1, 2) proliferation of KRT5+ cells at POD2, and 3) stratification and increased UPK expression at ≥ POD7^7,8^. Prior work revealed that the KRT5-lineage does not account for a majority of renal UPK+ cells formed following UUO, suggesting the existence of an alternative adult renal urothelium progenitor during UUO^15^. Therefore, we performed lineage analysis using *Upk2*^CreERT2^;*R26*^tdT^ mice to clarify whether renal UPK+ cells function as tissue repair progenitors, as is the case for UPK+ cells in bladder urothelium^17,19,20^. First, we inducibly and permanently labeled the UPK cell lineage with a tdTomato (TDT) reporter one week prior to UUO, a surgical model of UTO (Fig. 1a). Compared to adult kidneys at baseline, apical UPK3A expression was reduced at POD1; however, TDT expression remained (indelible tomato reporter), suggesting cells within the UPK-lineage persisted yet downregulated UPK expression (Fig. 1b). At POD7, these TDT+ cells were large and re-expressed UPK3A, suggesting post-obstruction UPK+ cells derive from the UPK-lineage. Next, we administered EdU at POD2 (at the peak of UUO-induced proliferation) to determine whether the TDT+ cells from the UPK-lineage could proliferate (Fig. 1a). At POD7, EdU was detected in TDT+;UPK3A+ cells (Fig. 1c), confirming that the UPK-lineage has the capacity to proliferate following UUO. Together these data demonstrate that UUO causes the UPK lineage to downregulate UPK expression (POD1), proliferate (POD2), and reacquire UPK (POD7). Altogether, these data indicate that UPK+ cells function as adult tissue repair progenitors during UUO, which aligns with roles for UPK+ intermediate cells in bladder urothelium repair^17,19,20^.

### scRNA-seq predicts PPARγ as a key transcription factor that regulates the UPK+ cell response to UUO.

To further characterize obstruction-induced renal urothelium remodeling, we analyzed publicly available scRNA-seq data from kidneys collected at baseline (POD0) and UUO at POD2, POD4, POD6, POD10, and POD14^35^. We first sorted cells based on UPK-lineage markers (*Upk1a, Upk1b, Upk2*, and *Upk3a*) and basal urothelial cell markers (*Krt5* and *Krt14*). We identified a pattern consistent with previous findings^6–8^, whereby Krt5+ and Krt14+ cell proportions increased following obstruction, while the proportion of UPK+ cells initially decreased (POD2), followed by their expansion beginning at POD4 (**Supplementary Fig 1a**). In addition to increased proportions of these urothelial cells, we observed matching increases in their gene expression at advanced UUO stages (**Supplementary Fig 1b**).

**Figure 2:**
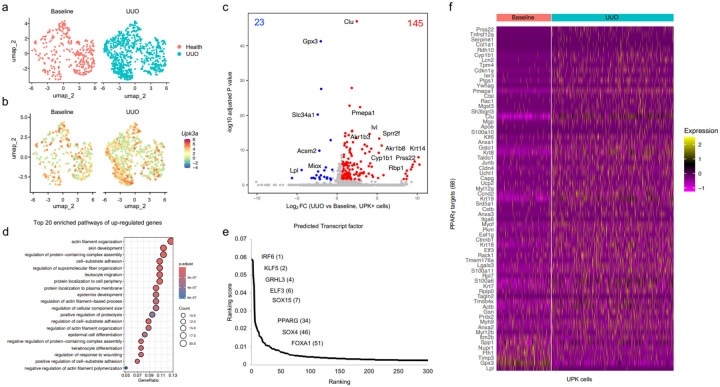
scRNA-seq demonstrates increased UPK cells and predicts PPARγ activation during UUO. **a** UMAP plots of scRNA-seq data showing 1,274 UPK-cells in baseline (318 cells) and UUO (956 cells) kidneys clustered by their expression of Upk1a, Upk1b, Upk2, and Upk3a. **b** UMAP plots of UPK-cells depicting Upk3a expression in single cells. **c** Volcano plot shows magnitude and significance of differentially expressed genes (DEGs) between baseline and UUO (POD6, POD10, POD14) K5-UCs. Blue: statistically significant downregulated genes in UUO (23); Red: statistically significant upregulated in genes in UUO (145). **d** Top 20 enriched biological processes of DEGs in UUO. **e** Graphs shows ChEA3 predicted transcription factors (TFs) ranked based on their predicted activity within differentially expressed genes in UUO compared to baseline. **f** Heatmap shows the expression of PPARγ target genes in baseline and UUO.

To better understand what regulates the increased expression of UPK following UUO, we compared UPK+ cells (*Upk1a+, Upk1b+, Upk2+*, and *Upk3a+*) at baseline (POD0) to advanced UUO stages (≥POD6) (Fig. 2a, b). We identified 168 differentially expressed genes (DEGs) in UPK+ cells of UUO compared to baseline kidneys, with 145 upregulated and 23 downregulated genes (Fig. 2c, **Supplementary Table 4**). Genes upregulated in UPK+ cells included *Lcn2, Clu, Krt5, Krt14*, and *Sox4*, highlighting increses in markers of kidney injury and progenitor activation. Gene ontology (**GO**) enrichment analysis revealed significant involvement in keratinocyte differentiation, epidermal cell differentiation, regulation of response to wounding, and actin filament organization, supporting observations that renal urothelium undergoes significant remodeling in response to UUO (Fig. 2d; **Supplementary Table 5**).

Next, we used DEGs to predict transcription factors that regulate the UPK+ cell response to UUO (**Supplementary Table 6**). Among the top ranked transcription factors, we identified KLF5, GRHL3, and ELF3, PPARγ, and FOXA1 (Fig. 2e). Interestingly, KLF5 is an upstream regulator of *Pparγ*, while *Grhl3, Elf3* and Foxa1 are downstream regulators of PPARγ signaling. Increased expression of PPARγ targets genes reveals increased activation of PPARγ during UUO, suggesting a key role for PPARγ in UPK+ cells. (Fig. 2f). Together these single cell data validate that UUO triggers renal urothelium remodeling that culminates in increased *Upk* expression and links the transcription factor PPARγ to UPK+ cells.

### PPARγ is induced and activated in UPK+ cells during UUO.

**Figure 3:**
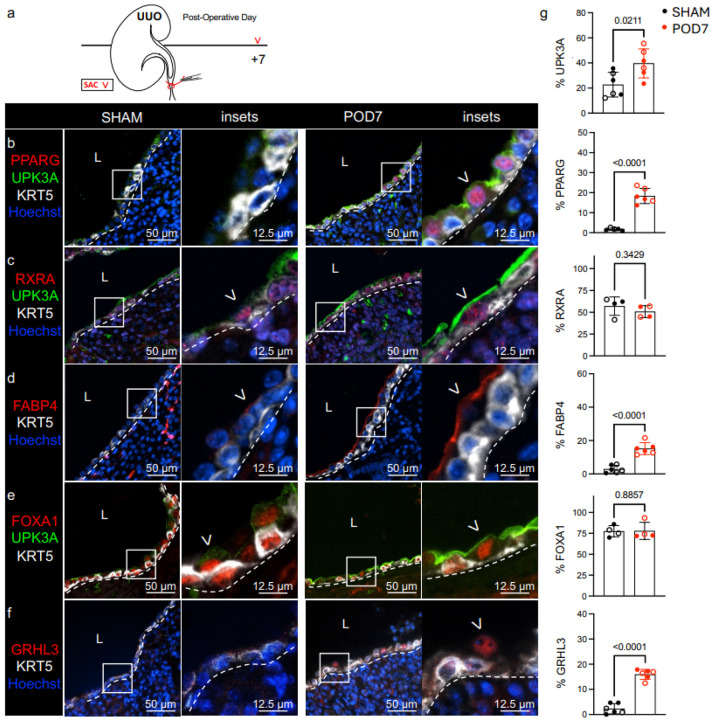
PPARγ is induced and activated in UPK+ cells during UUO. **a** Schematic depicts experimental plan. UUO: Unilateral Ureteral Obstruction; SAC: Sacrifice. **b** Representative micrographs and insets show anti-PPARG, -UPK3A, -KRT5, and Hoechst labeling in the renal urothelium of SHAM and POD7 kidneys. White dashed line: renal urothelium basement membrane; L: lumen; White arrowhead: PPARG+ UPK+ cell. **c**Representative micrographs and insets show anti-RXRA, -UPK3A, -KRT5, and Hoechst labeling in the renal urothelium of SHAM and POD7 kidneys. White dashed line: renal urothelium basement membrane; L: lumen; White arrowhead: RXRA+ apical cell. **d** Representative micrographs show anti-FABP4, -KRT5, and Hoechst labeling in the renal urothelium of SHAM and POD7 kidneys. White dashed line: renal urothelium basement membrane; L: lumen; White arrowhead: FABP4+ apical cell. **e** Representative micrographs and insets show anti-FOXA1, -UPK3A, and - KRT5 labeling in the renal urothelium of SHAM and POD7 kidneys. White dashed line: renal urothelium basement membrane; L: lumen; White arrowhead: FOXA1+ apical cell. **f**Representative micrographs and insets show anti-GRHL3, -KRT5, and Hoechst labeling in the renal urothelium of SHAM and POD7 kidneys. White dashed line: renal urothelium basement membrane; L: lumen; White arrowhead: GRHL3+ apical cell. **g** Graphs show (**top** to **bottom**) the area of UPK3A expression as a percent of the total renal urothelium area, the PPARG index as a percent of the total renal urothelium nuclei count, the RXRA index as a percent of the total renal urothelium nuclei count, the area of FABP4 expression as a percent of the total renal urothelium area, the FOXA1 index as a percent of the total renal urothelium nuclei count, and the GRHL3 index as a percent of the total renal urothelium nuclei count. SHAM (n=4–6 mice), POD7 (n=4–6 mice); filled circle: male; open circle: female. Bars: mean; Error bars: SD; *P*values: Unpaired Two-Tailed *t* test except for RXRA and FOXA1 where Mann-Whitney test was used due to nonparametric data distribution in POD7 cohort.

PPARγ promotes urothelial differentiation and UPK expression^21,22,36^, but a role for PPARγ has not been investigated in the renal urothelium. To investigate whether PPARγ promotes UPK expression during UUO, we first profiled UPK expression (using UPK1A, UPK1B and UPK3A) and components of the *Pparγ* pathway in baseline (SHAM-operated kidneys) and obstructed kidneys at POD7 (Fig. 3a and **Supplementary Fig. 2, 3**).

At baseline, the renal urothelium contains interspersed UPK+ cells, which express UPK1A, UPK1B, UPK2, and UPK3A^9^. scRNA-seq confirmed expression of *Upk1a, Upk1b, Upk2,* and *Upk3a* in the renal urothelium at baseline (**Supplementary Fig. 1**), and we confirmed the expression of UPK1A, UPK1B, and UPK3A (**Supplementary Fig. 2, 3**); however, antibody availability limited our ability to detect UPK2 (**data not shown**). As expected, UPK3A protein and *Upk3a* mRNA expression was significantly increased during UUO (POD7) compared to baseline (SHAM) (Fig. 3b**, c, e, g; Supplementary Fig. 3a, b**)^7^. Interestingly, PPARγ expression was low or undetectable in the renal urothelium at baseline, which is in stark contrast to high levels of PPARγ observed in bladder urothelium at baseline^21,22^. Instead, nuclear expression of PPARγ was significantly increased during UUO and localized to UPK3A+ cells (Fig. 3b, g). Next, we assessed the PPARγ binding partner, RXRa, which was expressed in the renal urothelium at equivalent levels at baseline and during UUO (Fig. 3c, g). To determine whether PPARγ was activated during UUO, we assessed FABP4, a direct transcriptional target of PPARγ, and found significantly increased apical expression of FABP4 in UPK3A+ cells (Fig. 3d, g; **Supplementary Fig. 3a**). FOXA1, a direct transcriptional target of PPARγ important for bladder urothelium differentiation, was expressed by KRT5+ and UPK3A+ cells at equivalent levels at baseline and during UUO. (Fig. 3e, g). Notably, GRHL3, a downstream target of PPARγ and transcriptional driver of urothelial differentiation, was significantly increased in the renal urothelium during UUO compared to baseline (Fig. 3f, g). Altogether, these data indicate that PPARγ is specifically expressed and activated in UPK+ cells during UUO and may regulate GRHL3 expression.

### Conditional deletion of PPARγ limits UPK expression during UUO.

**Figure 4:**
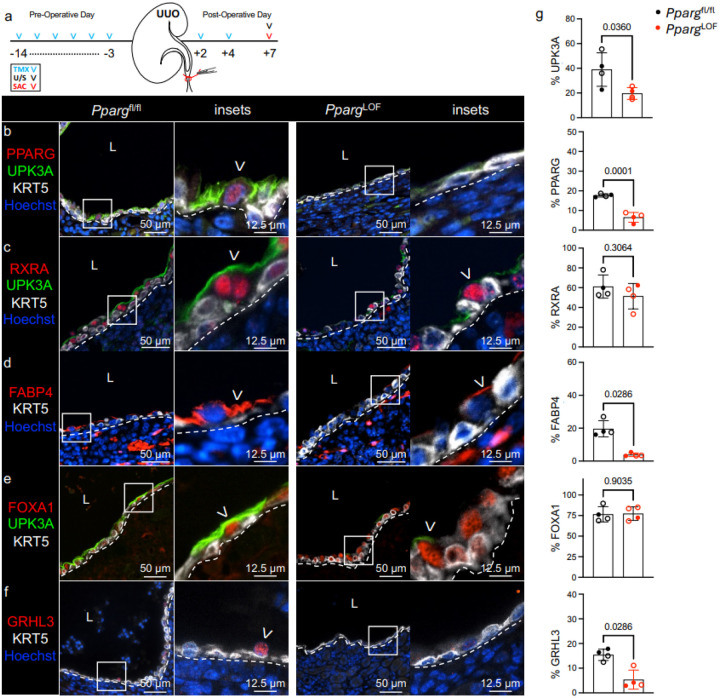
Conditional deletion of PPARγ limits UPK expression during UUO. **a** Schematic depicts experimental plan. UUO: Unilateral Ureteral Obstruction; TMX: Tamoxifen; U/S: Renal Ultrasound; SAC: Sacrifice. **b** Representative micrographs and insets show anti-PPARG, -UPK3A, -KRT5, and Hoechst labeling in the renal urothelium of *Pparg*^fl/fl^ and Upk2^CreERT2^;*Pparg*^fl/fl^ (*Pparg*^LOF^) kidneys at POD7. White dashed line: renal urothelium basement membrane; L: lumen; White arrowhead: PPARG+ UPK+ cell. **c** Representative micrographs and insets show anti-RXRA, -UPK3A, -KRT5, and Hoechst labeling in the renal urothelium of *Pparg*^fl/fl^ and *Pparg*^LOF^ kidneys at POD7. White dashed line: renal urothelium basement membrane; L: lumen; White arrowhead: RXRA+ apical cell. **d** Representative micrographs and insets show anti-FABP4, -KRT5, and Hoechst labeling in the renal urothelium of *Pparg*^fl/fl^ and *Pparg*^LOF^ kidneys at POD7. White dashed line: renal urothelium basement membrane; L: lumen; White arrowhead: FABP4+ apical cell. **e** Representative micrographs and insets show anti-FOXA1, -UPK3A, and -KRT5 labeling in the renal urothelium of *Pparg*^fl/fl^ and *Pparg*^LOF^ kidneys at POD7. White dashed line: renal urothelium basement membrane; L: lumen; White arrowhead: FOXA1+ apical cell. **f** Representative micrographs show anti-GRHL3, -KRT5, and Hoechst labeling in the renal urothelium of *Pparg*^fl/fl^ and *Pparg*^LOF^ kidneys at POD7. White dashed line: renal urothelium basement membrane; L: lumen; White arrowhead: GRHL3+ apical cell. **g** Graphs show (**top** to **bottom**) the area of UPK3A expression as a percent of the total renal urothelium area, the PPARG index as a percent of the total renal urothelium nuclei count, the RXRA index as a percent of the total renal urothelium nuclei count, the area of FABP4 expression as a percent of the total renal urothelium area, the FOXA1 index as a percent of the total renal urothelium nuclei count, and the GRHL3 index as a percent of the total renal urothelium nuclei count. *Pparg*^fl/fl^ (n=4 mice), *Pparg*^LOF^ (n=4 mice); filled circle: male; open circle: female. Bars: mean; Error bars: SD; *P*values: Unpaired Two-Tailed *t* test except for FABP4 and GRHL3 where Mann-Whitney test was used due to nonparametric data distribution in *Pparg*^LOF^ cohort.

To functionally evaluate the role of PPARγ in the renal urothelium during UUO, we generated *Upk2*^CreERT2^;*Pparg*^fl/fl^ (hereafter called *Pparg*^LOF^) and *Pparg*^fl/fl^ control mice. Mice were treated with TMX, received UUO, and were euthanized at POD7 (Fig. 4a). We observed a significant reduction in PPARγ in urothelium of *Pparg*^LOF^ mice during UUO (Fig. 4b, g; **Supplementary Fig. 4a**), while RXRa (PPARγ binding partner) remained unchanged (Fig. 4c, g). Next, we confirmed abrogation of PPARγ signaling as evidenced by a significant decrease in FABP4 (direct transcriptional target of PPARγ) in the renal urothelium of *Pparg*^LOF^ mice compared to *Pparg*^fl/fl^ (Fig. 4d, g; **Supplementary Fig. 3c**). FOXA1 (direct transcriptional target of PPARγ) expression was not affected by *Pparg*^LOF^ (Fig. 4e, g). However, GRHL3 (PPARγ downstream target) expression was significantly decreased in the renal urothelium of Pparg^LOF^ mice (Fig. 4f, g). This coincided with decreased UPK1A and UPK1B (**Supplementary Fig. 2b**), and significant decreases in UPK3A protein and Upk3a mRNA expression (Fig. 4b, c, e, g; **Supplementary Fig. 3c, d; Supplementary Fig. 4a**). Together these data indicate deletion of PPARγ abrogates GRHL3 and UPK expression in the renal urothelium during UUO.

### Conditional activation of PPARγ increases UPK expression during UUO.

**Figure 5:**
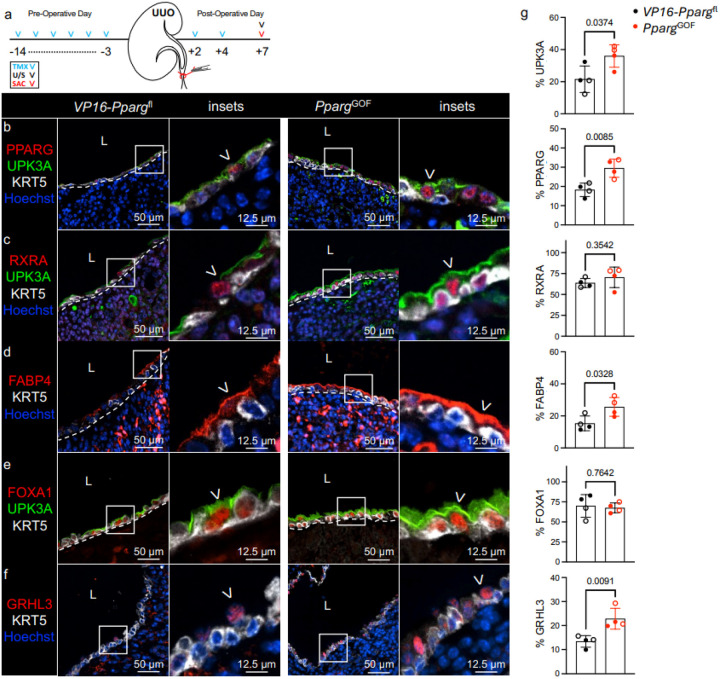
Conditional activation of PPARγ increases UPK expression during UUO. **a** Schematic depicts experimental plan. UUO: Unilateral Ureteral Obstruction; TMX: Tamoxifen; U/S: Renal Ultrasound; SAC: Sacrifice. **b** Representative micrographs and insets show anti-PPARG, -UPK3A, -KRT5, and Hoechst labeling in the renal urothelium of *VP16*-*Pparg*^fl^ (control) and *Upk2*^CreERT2^;*VP16-Pparg*^fl^ (*Pparg*^GOF^) kidneys at POD7. White dashed line: renal urothelium basement membrane; L: lumen; White arrowhead: PPARG+ UPK+ cell. **c** Representative micrographs and insets show anti-RXRA, -UPK3A, - KRT5, and Hoechst labeling in the renal urothelium of *VP16*-*Pparg*^fl^ and *Pparg*^GOF^ kidneys at POD7. White dashed line: renal urothelium basement membrane; L: lumen; White arrowhead: RXRA+ apical cell. **d** Representative micrographs and insets show anti-FABP4, -KRT5, and Hoechst labeling in the renal urothelium of *VP16-Pparg*^fl^ and *Pparg*^GOF^ kidneys at POD7. White dashed line: renal urothelium basement membrane; L: lumen; White arrowhead: FABP4+ UPK+ cell. **e** Representative micrographs and insets show anti-FOXA1, -UPK3A, and -KRT5 labeling in the renal urothelium of VP16-Pparg^fl^ and Pparg^GOF^ kidneys at POD7. White dashed line: renal urothelium basement membrane; L: lumen; White arrowhead: FOXA1+ apical cell. **f** Representative micrographs and insets show anti-GRHL3, -KRT5, and Hoechst labeling in the renal urothelium of *VP16-Pparg*^fl^ and *Pparg*^GOF^ kidneys at POD7. White dashed line: renal urothelium basement membrane; L: lumen; White arrowhead: GRHL3+ apical cell. Insets are also represented from each staining. **g** Graphs show (**top** to **bottom**) the area of UPK3A expression as a percent of the total renal urothelium area, the PPARG index as a percent of the total renal urothelium nuclei count, the RXRA index as a percent of the total renal urothelium nuclei count, the area of FABP4 expression as a percent of the total renal urothelium area, the FOXA1 index as a percent of the total renal urothelium nuclei count, and the GRHL3 index as a percent of the total renal urothelium nuclei count. *VP16-Pparg*^fl^ (n=4 mice), *Pparg*^GOF^ (n=4 mice); filled circle: male; open circle: female. Bars: mean; Error bars: SD; *P*values: Unpaired Two-Tailed *t* test.

To determine whether conditional activation of PPARγ could enhance UPK expression, we generated *Upk2*^CreERT2^;VP16-*Pparg*^fl^ (hereafter called *Pparg*^GOF^) mice. In *Pparg*^GOF^ mice, incorporation of a HSV VP16 activator fused to the Pparg1 N-terminal renders PPARγ conditionally activated in a ligand-independent manner^21^. *Pparg*^GOF^ and VP16-*Pparg*^fl^ control mice were treated with TMX, received UUO, and were euthanized at POD7 (Fig. 5a). We observed a significant increase in PPARγ in UPK3A+ cells of *Pparg*^GOF^ compared to *VP16-Pparg*^fl^ mice (Fig. 5b, g, **Supplementary Fig. 4b**). As was the case in *Pparg*^LOF^ experiments, RXRa (PPARγ binding partner) expression was unaffected by PPARγ activation (Fig. 5c, g). Next, we confirmed increased activation of PPARγ in the renal urothelium of *Pparg*^GOF^ mice as evidenced by a significant increase in apical FABP4 (direct transcriptional target of PPARγ) in UPK3A+ cells (Fig. 5d, g; **Supplementary Fig. 3e**). FOXA1 (direct transcriptional target of PPARγ) expression, remained unchanged with PPARγ activation in the renal urothelium (Fig. 5e, g). Instead, GRHL3 (PPARγ transcriptional target) was significantly increased in the renal urothelium of *Pparg*^GOF^ mice compared to *VP16-Pparg*^fl^ during UUO (Fig. 5f, g). This coincided with increased UPK1A and UPK1B (**Supplementary Fig. 2c**), and significant increase in UPK3A protein and *Upk3a* mRNA expression (Fig. 5b, c , e, g; **Supplementary Fig. 3e, f; Supplementary Fig. 4b**). These data indicate that conditional and constitutive activation of PPARγ increases UPK expression in the renal urothelium during UUO.

### PPARγ activation in renal urothelium promotes renal structural integrity during UUO.

**Figure 6:**
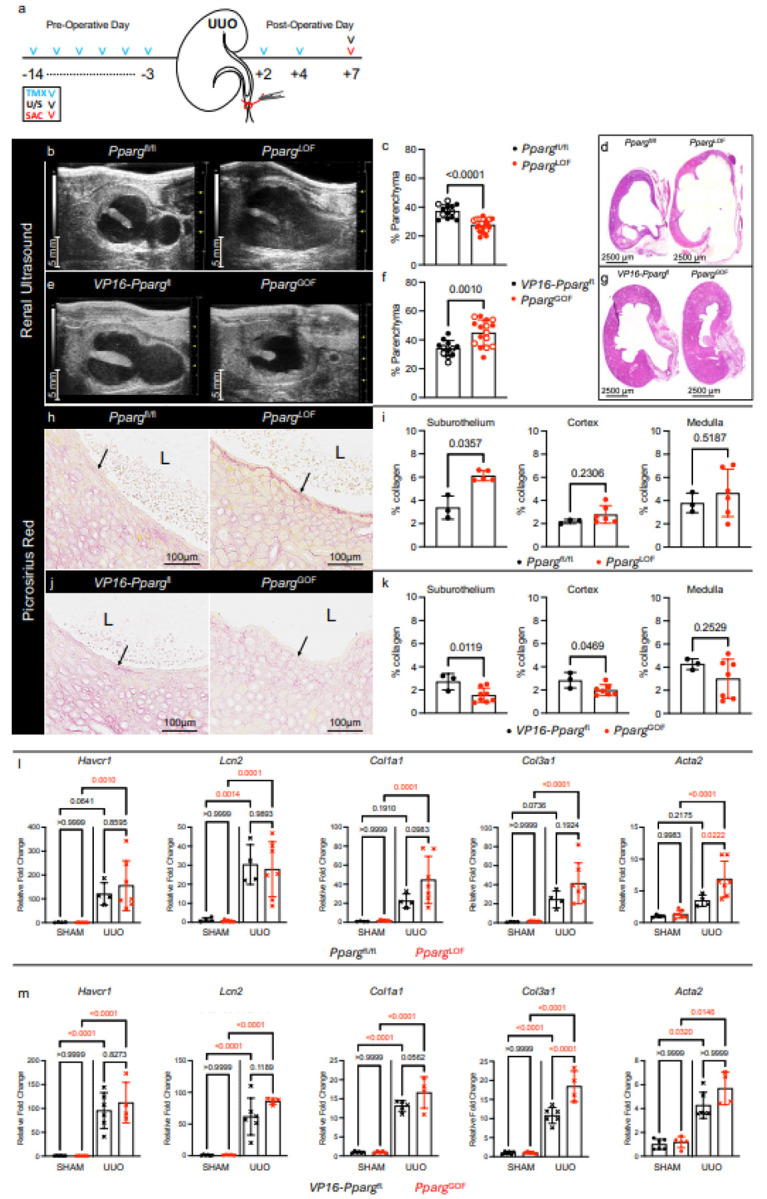
PPARγ activation in renal urothelium promotes renal structural integrity during UUO. **a** Schematic depicts experimental plan. UUO: Unilateral Ureteral Obstruction; TMX: Tamoxifen; U/S: Renal Ultrasound; SAC: Sacrifice. **b** Representative sonograms of *Pparg*^fl/fl^ and *Pparg*^LOF^ kidneys at POD7. **c** Graph shows the fractional parenchyma percentage. *Pparg*^fl/fl^ (n=9 males, n=4 females), *Pparg*^LOF^ (n=13 males, n=7 females); filled circle: male; open circle: female. Bars: mean; Error bars: SD; *P*values: Unpaired Two-Tailed t test. **d** Representative micrographs of H&E-stained kidney sections from *Pparg*^fl/fl^and *Pparg*^LOF^ POD7 kidneys. **e** Representative sonograms of *VP16-Pparg*^fl^ and *Pparg*^GOF^ kidneys at POD7. **f** Graph shows the fractional parenchyma percentage. *VP16-Pparg*^fl^ (n=7 males, n=5 females), *Pparg*^GOF^ (n=8 males, n=8 females); filled circle: male; open circle: female. Bars: mean; Error bars: SD; *P*values: Unpaired Two-Tailed t test. **g** Representative micrographs of H&E-stained kidney sections from *VP16*-*Pparg*^fl^ and *Pparg*^GOF^ POD7 kidneys. **h** Representative micrographs show Picrosirius Red (PSR) stain in *Pparg*^fl/fl^ and *Pparg*^LOF^ kidneys at POD7, captured using standard brightfield light. L: lumen; Arrows: suburothelium collagen. **i** Graphs show the collagen area (measured using polarized light micrographs) as a percent of the suburothelium compartment, renal cortex, and renal medulla areas. *Pparg*^fl/fl^ (n=3 mice), *Pparg*^LOF^ (n=5 mice). Bars: mean; Error bars: SD; *P*value: Unpaired Two-Tailed *t* test except Suburothelium graph where Mann-Whitney test was used due to nonparametric data distribution in *Pparg*^LOF^ cohort. **j** Representative micrographs show Picrosirius Red (PSR) stain in VP16-*Pparg*^fl^ and *Pparg*^GOF^ kidneys at POD7, captured using standard brightfield light. L: lumen; Arrows: suburothelium collagen. **k** Graphs show the collagen area (measured using polarized light micrographs) as a percent of the suburothelium compartment, renal cortex, and renal medulla areas. *VP16*-*Pparg*^fl^ (n=3 mice), *Pparg*^GOF^ (n=8 mice). Bars: mean; Error bars: SD; *P*value: Unpaired Two-Tailed *t* test. **l-m** Graphs show the relative fold change of Havcr1, Lcn2, Col1a1, Col3a1, and Acta2 mRNA expression in SHAM and UUO (POD7) kidneys from *Pparg*^fl/fl^ and *Pparg*^LOF^ mice (**l**), and *VP16-Pparg*^fl^ and *Pparg*^GOF^ mice (**m**). Bars: mean; Error bars: SD; *P* values: One Way Anova with Šídák’s multiple comparisons test.

Prior studies discovered that the urothelial plaque (formed by UPK proteins) promotes renal structural integrity^7^. Since our findings demonstrate that PPARγ-regulates UPK expression, we evaluated the impact of conditional PPARγ deletion and activation on kidney structure during UUO at POD7 (Fig. 6a). Using renal ultrasound to capture images of the longitudinal axis of the kidney and histologic examination, we observed that the renal parenchyma was significantly thinned in *Pparg*^LOF^ mice compared to *Pparg*^fl/fl^ mice at POD7 (Fig. 6b–d, **Supplementary Fig. 5**). In contrast, *Pparg*^GOF^ mice had significantly more renal parenchyma compared to *VP16-Pparg*^fl^ mice at POD7 (Fig. 6e–g, **Supplementary Fig. 5**). Importantly, we did not observe renal structural defects in these mice prior to UUO (**Supplementary Fig. 4c, d**). Although the link between renal urothelium and parenchymal integrity remains unclear, we measured collagen deposition using Picrosirius red (**PSR**) stained sections to assess extracellular matrix accumulation in each experimental group.

We observed a significant increase in collagen beneath the renal urothelium (suburothelium collagen) in Pparg^LOF^ (Fig. 6h, i), and a significant decrease in suburothelium collagen in *Pparg*^GOF^ mice (Fig. 6j, k). While the interstitium immediately beneath the urothelium was affected by PPARγ manipulation, collagen deposition in the cortical and medullary compartments was less affected (Fig. 6i, k). Since the UUO model is not directly amenable to renal functional measurements, we profiled markers of kidney injury *Havcr1* (encodes KIM-1) and *Lcn2* (encodes Ngal), and extracellular matrix markers *Col1a1* (encodes alpha-1 type I collagen), *Col3a1* (encodes alpha-1 type III collagen), and Acta2 (encodes smooth muscle alpha (α)-2 actin) using RT-qPCR in whole kidneys. As expected, UUO kidneys expressed higher levels of *Havcr1, Lcn2, Col1a1, Col3a1*, and *Acta2* compared to sham-treated kidneys; however, we did not find significant exacerbation or protection with conditional PPARγ deletion or activation, respectively (Fig. 6l, m). Together these data indicate that conditional activation of PPARγ in the renal urothelium promotes renal structural integrity during UUO, but does not exert widespread protection for the obstructed kidney.

## Discussion

Congenital and acquired UTO cause bladder-like renal urothelium remodeling which culminates in increased UPK+ cells and UPK expression^6–8^. Deletion of UPK+ cells or the UPK-containing urothelial plaque exacerbates parenchymal thinning and accelerates renal dysfunction in acquired and congenital UTO, respectively. Therefore, renal urothelium remodeling and increased UPK represent protective adaptations during UTO^7^, but the cellular and molecular bases underlying renal urothelial remodeling were unclear. In this study, we closed key knowledge gaps. First, we established that adult UPK+ cells are tissue repair progenitors. Second, we demonstrated that PPARγ promotes GRHL3 and UPK expression in the renal urothelium. Finally, we demonstrated that cell-specific activation of PPARγ promotes renal structural integrity during UTO. These findings enhance our understanding of the renal urothelium and form the foundation for the development of therapies aimed at increasing UPK expression to preserve renal parenchyma.

### UPK+ cells are tissue repair progenitors during UUO.

Unlike high turnover epithelia found in the skin and gut, urothelium is nearly quiescent during homeostasis^37–39^, which complicates fate mapping analysis. However, we previously reported that KRT5+ cells display a temporally restricted potential to form UPK+ cells, such that embryonic (E17-E18), neonatal (P1-P7) and juvenile (P14) KRT5+ cells give rise to UPK+ cells, while adult KRT5+ cells are lineage restricted^15,16^. Mechanical, chemical, and bacterial injuries engage robust proliferation to quickly restore damaged urothelium^17,40–46^. In the kidney, congenital and acquired UTO engage a conserved program of renal urothelium remodeling that culminate in increased UPK expression. Here, we used UUO, a surgical UTO model, to precisely control the timing of obstruction and study the cellular and molecular basis of renal urothelium remodeling and publicly available single cell RNAseq data that spans several UUO timepoints. Notably, UUO elicits 1) loss of UPK expression at post-operative day (POD)1, 2) KRT5+ cell expansion at POD2, and 3) increased apical UPK expression and expansion of UPK+ cells at ≥ POD7^7,8,15^. While loss of UPK expression and KRT5+ cell expansion suggests that KRT5+ cells may function as tissue repair progenitors during UTO, prior lineage analysis concluded that KRT5+ cells did not account for the majority of UPK+ cells following UUO^15^. Remarkably, while UPK3A expression is diminished at POD1, we observed that the UPK-lineage remains intact, proliferates, and forms apical UPK+ cells at POD7. Consistent with bladder urothelium tissue repair studies, our findings demonstrate an important role for renal UPK+ cells during UUO^17,19,20^.

### Activation of urothelial PPARγ promotes UPK expression during UUO.

Our bioinformatics examination predicted activation of PPARγ in UPK+ cells during UUO. PPARγ promotes a program of urothelial differentiation in vitro and in vivo that culminates in increased UPK expression^21–26^. Upon ligand binding, the PPARγ:RXRa complex regulates downstream targets by binding to PPREs^22,28^. While RXRa expression remains consistent, we show that PPARγ is induced and activated in renal UPK+ cells only during UUO. We also determined that PPARγ is indispensable for UUO-induced urothelial differentiation since UPK was reduced in *Pparg*^LOF^ mice and increased in *Pparg*^GOF^ mice during UUO. These observations are consistent with recent in vivo studies in bladder urothelium where conditional deletion of PPARγ was shown to abrogate terminal differentiation and conditional activation of PPARγ was shown to promote basal urothelial cell differentiation^21,22^.

We used FABP4 to assess PPARγ activation, and deletion in our studies. While FABP4 is induced during UUO and affected by genetic manipulation of PPARγ, it is unclear whether FABP4 orchestrates a program of urothelial differentiation. Additional direct downstream targets of PPARγ in urothelium include FOXA1, and IRF1, which are known to promote UPK1A, UPK2, and UPK3A expression^25^. While we were unable to detect IRF1 (**data not shown**), we found that FOXA1 was expressed by renal urothelium at baseline and following UUO and did not appear to be affected by genetic manipulation of PPARγ. Therefore, it is unlikely that UUO-induced renal urothelium differentiation occurs through a PPARγ:FOXA1-dependent manner. GRHL3 is a PPARγ downstream regulator and is reduced in PPARγ-deficient bladder urothelium^22^. GRHL3 promotes urothelial differentiation and selectively binds to the *Upk2* promoter^29^. Our findings reveal that GRHL3 is induced during UUO and affected by genetic manipulation of PPARγ. Together these findings support a role for PPARγ:GRHL3 in UUO-induced renal urothelial differentiation. While additional studies are required to functionally validate a role for GRHL3 in renal urothelium differentiation, additional PPARγ downstream regulators must also be investigated.

### Activation of urothelial PPARγ promotes parenchymal integrity during UUO.

We observed parenchymal loss in UPK-deficient *Pparg*^LOF^ kidneys, and parenchymal preservation in UPK-enhanced *Pparg*^GOF^ kidneys during UUO. These findings align with our prior work which established a critical role for UPK in promoting structural integrity within the obstructed kidney^7^. However, the mechanism by which UPK expression impacts parenchymal integrity remain unclear. One possibility is that the UPK plaque promotes cellular compliance^12^; therefore, increased UPK may lead to better accommodation of increased urine volumes during UTO. A second possibility is that the UPK plaque promotes a water-tight urothelial barrier^10–12^; therefore increased UPK may limit urine permeability during UTO. A third possibility stems from anti-inflammatory and anti-fibrotic roles for PPARγ in numerous organ systems, including the kidney (reviewed in^47^). Indeed, our findings reveal increased suburothelial collagen in obstructed *Pparg*^LOF^ kidneys, while *Pparg*^GOF^ kidneys were characterized by reduced suburothelial collagen. Pharmacologic activation of PPARγ by anti-diabetic thiazolidinediones limit inflammation and fibrosis during UTO. Kawai et al. demonstrated that troglitazone treated mice exhibit reduced transforming growth factor beta 1 (TGF-b1), interstitial alpha smooth muscle actin (aSMA) and collagen I during UUO^48^, revealing that PPARγ activation suppresses interstitial inflammation and fibrosis. More recently, Wei et al. used ultrasound guided delivery of rosiglitazone loaded nanoparticles to show that PPARγ activation reduced collagen deposition and attenuated interstitial fibrosis during UUO in rats^49^. Importantly, our findings which reveal altered UPK and suburothelial collagen levels in obstructed *Pparg*^LOF^ and *Pparg*^GOF^ kidneys highlight the significance of a urothelial-specific contribution to UTO-induced collagen deposition. Future studies are warranted to dissect the interplay between urothelial PPARγ activation and renal compliance, urothelial barrier function and inflammation during UTO.

### Endogenous Regulation and Activation of PPARγ.

Several transcription factors regulate *Pparγ* (reviewed in^50^). In urothelium, Kruppel-like transcription factor 5 (KLF5) and the transcriptional regulator Brahma-related gene 1 (BRG1) are shown to be upstream of PPARγ and their deficiency *in vivo* alters UPK expression in the bladder and ureter, respectively^27,36^. Additional studies are needed to clarify the transcriptional regulation of *Pparγ* in renal urothelium following UTO.

PPARγ activation occurs predominantly through a ligand-dependent manner. Endogenous ligands include fatty acids and eicosanoids, such as the prostaglandin (PG) metabolite 15-Deoxy-Δ -^12,14^-Prostaglandin J2 (**15d-PGJ**_2_)^51^. 15d-PGJ2 is a terminal metabolite derived from cyclooxygenase (**COX**)-2 metabolism of arachidonic acid (reviewed in^52^). 15d-PGJ2 is present in human urine^53^, but it is unclear whether urinary 15d-PGJ2 levels are increased in obstructed kidneys. Furthermore, COX-2 is upregulated in bladder urothelium during tissue repair^54,55^, but it is unclear whether 15d-PGJ2, or COX-2 are impacted or relevant to UTO-induced renal urothelium remodeling. Additional studies are needed to clarify how PPARγ is endogenously activated in the renal urothelium.

In conclusion, our data show that PPARγ promotes UPK expression in the renal urothelium, and that activation of PPARγ in the renal urothelium promotes renal structural integrity during UUO. Further studies are required to delineate events upstream and downstream of PPARγ, and whether activation of PPARγ in the obstructed kidney can serve as a therapeutic measure to mitigate parenchymal injury and preserve renal function after unobstruction. Finally, this study compels an investigation of PPARγ in reversible UUO and models of congenital urinary tract obstruction, such as the Megabladder mouse^56^, where the impact of urothelial PPARγ manipulation on kidney function can be investigated.
